# Isothermal Pyrolysis of Bamboo and Pinewood Biomass: Product Characterization and Comparative Study in a Fluidized Bed Reactor

**DOI:** 10.3390/bioengineering12020099

**Published:** 2025-01-22

**Authors:** Manqoba Shezi, Sammy Lewis Kiambi

**Affiliations:** 1 Green Energy Research Group, Department of Chemical Engineering, Durban University of Technology, Durban 4000, South Africa; 2 Chemical Engineering Department, Vaal University of Technology, Vanderbilpark 1911, South Africa

**Keywords:** biomass, bio-oil, fast pyrolysis, biofuels

## Abstract

Fast pyrolysis of biomass is crucial for sustainable biofuel production, necessitating thorough characterization of feedstocks to optimize thermal conversion technologies. This study investigated the isothermal pyrolysis of bamboo and pinewood biomass in a sand-fluidized bed reactor, aiming to assess biomass suitability for commercial bio-oil production. The pyrolysis products and biomass species were characterized through proximate and ultimate analyses, along with GCMS, FTIR, SEM/EDX, and structural analysis to assess their chemical and physical properties. Results indicated that pine bio-oil possesses superior energy density, with a higher calorific value (20.38 MJ/kg) compared to bamboo (18.70 MJ/kg). Pine biomass yielded greater organic phase bio-oil (BOP) at 13 wt%, while bamboo produced 9 wt%. Energy yields were also notable, with pine exhibiting an energy yield of 15% for bio-oil organic phase (EBOP), compared to 11% for bamboo. The fibrous nature of bamboo biomass resulted in less-reacted biomass at constant reaction time due to flow resistance during pyrolysis. Pine bio-oil organic phase (P-BOP) demonstrated a higher heating value (23.90 MJ/kg) than bamboo (B-BOP). The findings suggest that while both biomass types are viable renewable energy sources, pine biomass is more favorable for commercialization due to its superior energy properties and efficiency in pyrolysis.

## 1. Introduction

The significant environmental degradation caused by climate change, mainly due to extensive fossil fuel consumption, has prompted increased interest in bio-based fuels as potential alternatives. This shift aims to decrease dependence on fossil fuels overall [1,2]. As a result, it has become evident that energy should be renewable, cost-effective, convenient, safe, and sustainable. Nowadays, it is considered feasible and promising to convert biomass via thermochemical processes into petrochemicals and biofuels [3]. These processes use various biomasses that can be processed into a wide range of products, offering great productivity. While virtually all plant biomass can undergo pyrolysis, certain species exhibit more excellent suitability than others under similar operational conditions [4]. Energy crops such as bamboo and giant reed have gained enormous exploration for future energy production due to their high economic value, short rotation, high productivity, and improvement for sustainable management. Bamboo is considered invasive in other parts of the world, especially the running type bamboo; thus, its application in biofuel production is vital for the efficient use of marginal lands [5]. Currently, there is no effective control measure for dealing with the extensive amount of invasive energy crops. Incinerating it directly is the adopted technique to dispose of it; however, this results in the release of harmful gases, which are carbon monoxide, carbon dioxide, nitrogen oxides, and fine particulate matter [6]. The attempts to apply biological and burning methodologies to control the invasiveness of the non-food energy crops add no value compared to utilizing the plants for bioenergy generation. Patel and Kumar [7] investigated the pyrolysis of gigantic reed grass using thermogravimetry/mass spectrometry (TG-MS). The authors concluded that energy grass has a stronger thermochemical reactivity and a quicker devolatilization. Woody biomass, on the other hand, is known for its low ash content when compared to energy crops. Pinewood is among the most abundant biomass sources in the world, and extensive work has been done in producing bio-oil and biochar via thermochemical conversion technologies [8,9,10,11,12,13,14,15]. In forested areas, logs are often harvested for fuelwood or incinerated in open settings once dry to clear space for alternative forest activities. Wood shavings and residuals from sawmills are similarly combusted as a method of disposal [16]. The emissions resulting from combustion exacerbate environmental challenges related to climate change. Optimal utilization of biomass for bioenergy production holds considerable promise in addressing global energy demands and economic issues in developing countries. In thermochemical conversion, the primary techniques include pyrolysis, liquefaction, gasification, and combustion [17]. Among the aforementioned thermochemical techniques, pyrolysis is the preferred approach, as it is cost-effective and easy to use for the production of biofuels and petrochemicals from biomass. This technique is categorized into slow, fast, and flash pyrolysis [18]. The choice of pyrolysis type to employ depends on the desired product distribution. Fast pyrolysis is a good choice when high bio-oil yields are desired, while slow and flash are selective to higher yields of biochar and biogas, respectively. Fast pyrolysis of biomass represents a pivotal avenue for biofuel production, recognized as environmentally viable for future energy needs. Comprehensive knowledge of biomass pyrolysis characterization and its derived products is essential for the design of thermal conversion technology systems and for selecting appropriate feedstock species for efficient biofuel production [19]. Therefore, thorough characterization of the parent feedstock is fundamental in pyrolysis technology. While extensive research has been conducted on the pyrolysis of bamboo and pine biomass individually, limited studies have examined the pyrolysis of these materials when continuously fed into a reactor for bulk bio-oil production. This is particularly critical for advancing large-scale bio-oil production, yet this area has largely been overlooked in the existing literature. In particular, the effects of fibrous feedstocks, such as bamboo, in continuous feeding setups remain underexplored. In the current work, two biomass species were characterized and pyrolyzed in a sand-fluidized bed reactor to determine their suitability for commercial bio-oil production. Other pyrolysis products were also characterized to assess their potential as sources of bioenergy. The comparative isothermal fast pyrolysis study of cane-based biomass and wood-based biomass for the production and characterization of bio-oil in a sand-fluidized bed reactor has not been done to the best of the authors’ knowledge. This study uses *N. Henon* bamboo, a highly invasive species that has not been widely examined in thermochemical conversion applications. In contrast, *Moso* bamboo is the most commonly studied species [20,21,22,23,24,25,26,27,28]. The introduction of *N. Henon* bamboo as a feedstock for pyrolysis represents a novel approach in biomass research. The key research question guiding this study is: How does the pyrolysis behavior of bamboo compare to pine biomass under similar conditions? This study addresses these gaps by conducting a comprehensive analysis of bamboo and pine biomass under identical pyrolysis conditions, with a focus on the impact of feedstock properties on bio-oil yield and composition.

## 2. Materials and Methods

### 2.1. Sample Harvesting and Preparation

Bamboo (*Phyllostachys nigra “Henon”*) and pinewood biomass were obtained from Auburn University, USA. Bamboo was harvested during summer, having a height of 4–6 m and a diameter of 5 cm. The pinewood considered in this study was longleaf pine (*Pinus palustris*) and was felled, debarked, and ground into small chips to be stored in the biomass storage warehouse. In preparation for pyrolysis experiments, biomass was dried for 24 h at 105 °C. The oven-dried biomass was ground by a hammer mill (C.S. Bell Co., model 10HBLPK, Tiffin, OH, USA) prior to sieving. A sieve shaker was used to obtain the desired particle size range of 0.6–1 mm.

### 2.2. Biomass Characterization

Elemental analysis (CHNS) was performed using an Elementar Vario Micro Select, according to ASTM D5373 [29]. Biomass moisture content and ash content were determined according to ASTM D4442-07 [30] and E1755-01 [31], respectively. The volatiles were determined according to ASTM E872 [32], and fixed carbon content was determined by difference. A thermolyne (Thermo Fisher Scientific, Auburn, AL, USA) furnace was used during the oxidation process. Thermal degradation behavior of biomass was carried out using a thermogravimetric analyzer (TGA5500/Discovery Series) under a nitrogen atmosphere for a temperature range of 25–800 °C at a heating rate of 10 °C/min. Platinum HT pan types were used for sample loading, and a ramp procedure was employed. Lignin and structural carbohydrates were estimated by following the National Renewable Energy Laboratory method (NREL) [33,34,35]. Briefly, 10 g of biomass was loaded into the extraction crucible for a sequential water and ethanol extraction process. After extraction, 0.3 g of biomass was loaded into pressure tubes to conduct acid hydrolysis using 72% sulfuric acid, diluted with deionized water, and autoclaved for 1 h. Vacuum filtration was used to separate solids and liquids using filtering crucibles. The solid residue was heated to 105 ± 5 °C to determine the dry residue weight, followed by oxidation at 575 ± 25 °C to determine Klason lignin (AIL). Using the filtrate, the acid-soluble lignin (ASL) was determined using a UV/vis spectrophotometer (Thermo Scientific, Orion Aquamate AQ8100). Sugars were analyzed using high-performance liquid chromatography (HPLC) (Agilent Technologies 1260, Santa Clara, CA, USA) using the BioRad HPX-87P column. The chemical functional groups of biomass were identified by using Fourier transform infrared spectroscopy (FTIR) at a range of 4000–400 cm^−1^. The spectra were generated using the FTIR-ATR, Thermo Fisher Scientific Smart ITX (Nicolet iSO10, Waltham, MA, USA) spectrometer.

### 2.3. Fast Pyrolysis with Fluidized Bed Reactor

**Figure 1:**
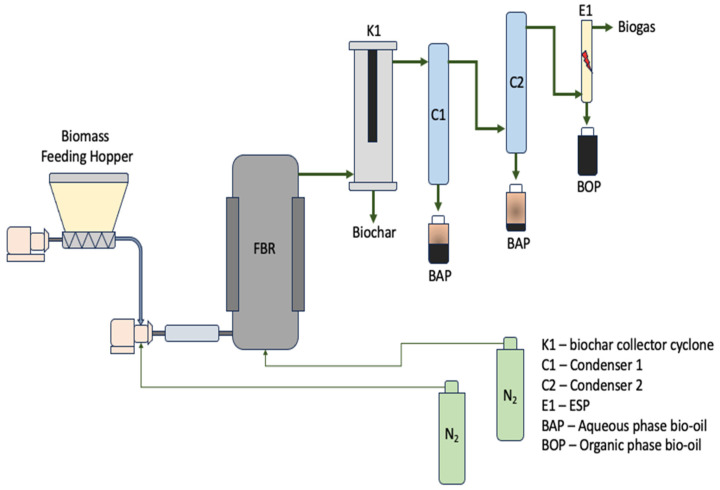
Schematic diagram for fast pyrolysis in a fluidized bed reactor (FBR).

The fast pyrolysis runs were conducted using a fluidized gas-solid reactor developed by the Bioenergy Laboratory of Auburn University (Auburn, AL, USA), as shown in Figure 1. Pinewood or bamboo biomass was loaded into the hopper. White fine silica sand (1235 ± 0.5 g) was used to fluidize the bed and enhance the heat transfer within the reactor. The sand was sieved to a desired particle size of 0.3–0.22 mm and then was loaded into the reactor. The pyrolysis system is comprised of a hopper (screw auger/airlock feeding), fluidized bed reactor (FBR), hot filtration unit, and condensing system that is equipped with an electrostatic precipitator (ESP). The reactor and char filter were preheated to the desired temperature before starting each run using electrical heaters. Once the desired temperatures were reached, the system was purged with nitrogen gas for 13 ± 2 min to ensure an inert atmosphere. Two nitrogen lines with flow rates of 34 standard liters per minute (SLPM) and 6 ± 1 SLPM for the reactor and backflow prevention were used, respectively. The reactor temperature was allowed to reach 595 ± 18 °C before feeding the biomass. Then biomass was conveyed into the reactor at a rate of 27 g/min by a screw auger that is mechanically controlled by the weigh feeder controller (C-702, ACRISON, Moonachie, NJ, USA). Each pyrolysis run was allowed to run for 2 h at a temperature of 544 ± 33 °C in replicates of three. The reactor, pipelines, and char filter were properly insulated to minimize any heat loss to the surroundings that can result in undesired condensation taking place before reaching the cooling system. The cooling medium was ethylene glycol at 2 ± 0.5 °C and 1.2 bar with a pumping capacity flow rate (PCF) of 40 L/min from a recirculating chiller (Julabo, Allentown, PA, USA). Non-condensable gases were collected in 1 L Tedlar bags (RESTEK, scenic Centre County, PA, USA) after 20 min of starting the pyrolysis reaction for compositional analyses. Biochar was collected and weighed. The sand and biochar balance was used to correctly determine the amount of biochar obtained from the char filter and the reactor. The total weight of biogas was determined by mass balance after the pyrolysis reaction. The vapors from the reactor passed through the char filter, where biochar was collected. Thereafter, the liquid product (aqueous phase) was condensed by two condensers in series. The organic phase bio-oil was precipitated from the non-condensable gases (NCGs) using one ESP. Bio-oil was continuously obtained and weighed for the duration of the reaction using Nalgene bottles, style 2104 (B9157 and B9407, Sigma-Aldrich, St. Louis, MO, USA), for sample collection. The bio-oil collected from the ESP was classified as the bio-oil organic phase (BOP) and was used for hydrotreating experiments. The percentage yields of the bio-oil organic phase (BOP%), aqueous phase (BAP%), biochar (BC%), and pyrolysis gas (BG%) were calculated by the following equations:

(1) $$BOP\%=\frac{m_{bop}}{m_{bm}}\times 100$$

(2) $$BAP\%=\frac{m_{bap}}{m_{bm}}\times 100$$

(3) $$BC\%=\frac{m_{bc}}{m_{bm}}\times 100$$

where m_bop_, m_bm_, m_bap_, and m_bc_ are the masses of the organic phase, biomass, aqueous phase, and biochar, respectively. The principle of mass conservation was used to determine the biogas production yield according to the following equation:

(4) $$BG\%=100-BC\%-\left(BOP\%+BAP\%\right)$$

### 2.4. Product Characterization

Elemental analysis (CHNS) was performed using an Elementar Vario Micro Select, according to ASTM D5373 [29] and ASTM D5291 [36]. The oxygen content was determined by difference, and the results were reported on a dry basis. The moisture content of bio-oil was measured by Karl Fischer titration by using Aquastar (combititrant 5 keto, volumetric KFT for aldehydes and ketones) as a titrator/reagent and Apura (combi-solvent keto, volumetric KFT, ca. 5 mg H_2_O) as a solvent. Total acid number (TAN) was measured by a Mettler T50 autotitrator using a total acid number titration solvent mixture and 0.1 M KOH 2-propanol as the titrating reagent (titrant), according to ASTM D664-07 [37]. SEM/EDX was used to determine the inorganic contents/elements of biochar. A bomb calorimeter (IKA C200 calorimeter, IKA Works, Wilmington, NC, USA) was used to determine the higher heating values of biomass and bio-oil organic phase. The ash content was determined according to ASTM D 482 [38], where a BOP sample was ignited and allowed to combust until only carbon material and ash (inorganics) remained in the vessel. In order to reduce the carbonaceous residue to ash, the ignited sample was heated at 775 ± 25 °C for 20 min in a muffle furnace. Following oxidation, the sample was cooled to room temperature in a desiccator and then weighed. The viscosity (kinetic and dynamic) and density were measured using the Automatic Kinematic Viscometer (Anton Paar Instrument: SVM, Ashland, OR, USA) at 40 °C. Biogas chemical composition was analyzed using the Agilent 490 4-channel Micro GC, with helium and argon as carrier gases for different channels. The calibration curves were initiated using a standard gas mixture of hydrogen (H_2_), carbon monoxide (CO), carbon dioxide (CO_2_), and methane (CH_4_). The “others (C_x_H_y_)” on the biogas balance was obtained by difference, i.e., non-quantified gases were classified as others. The chemical composition of organic phase bio-oil was analyzed by gas chromatography coupled with a mass spectrometric detector (GCMS). The experiments were carried out on an Agilent 5977C GC/MSD with helium gas as a carrier gas. The National Institute of Standards and Technology (NIST) spectral library was used to identify the chemical compounds. Briefly, the samples were weighed and then diluted with 2 mL of methanol. The diluted sample was injected, and the inlet temperature was set to 280 °C at a split ratio of 10:1. The column temperature was held at 50 °C for 5 min, then heated to 280 °C at a ramping rate of 10 °C/min with a 5 min holding time. Thermal behavior of the spent catalyst was used to examine carbon deposition on the catalyst. The TG-50H detector with an alumina cell was used for examination at a heating rate of 10 °C/min and a holding temperature of 800 °C (2 min) under the air atmosphere (20 mL/min).

## 3. Results and Discussion

### 3.1. Biomass Characterization: Proximate and Ultimate Analysis

The characterization results from the proximate and ultimate analyses provide valuable insights into the suitability of bamboo and pine as feedstocks for pyrolysis-based bio-oil production (Table 1). Proximate analysis reveals critical parameters such as moisture, volatile matter, ash, and fixed carbon contents, which significantly influence pyrolysis behaviour and product distribution. Bamboo demonstrates a lower moisture content (4.01 wt%) compared to pine (6.76 wt%), indicating a higher energy efficiency. Pine, however, exhibits higher volatiles (92.50 wt%) compared to bamboo (85.90 wt%), suggesting a greater potential for bio-oil yield. Moreover, bamboo presents a higher fixed carbon content (12.13 wt%) compared to pine (7.17 wt%), indicating a propensity for more stable pyrolysis reactions and potentially higher biochar yields. The 41% decline in fixed carbon for pine biomass was attributed to low ash content and high volatiles. Bamboo is nutrient rich and tends to absorb minerals from the ground, which results in high inorganics that contribute to high ash content, as shown by a 2% ash content compared to 0.3% for pine. Jung, Kang [39] reported an ash content of 1.7 wt% for bamboo, while Kato, Kohnosu [40] found the ash to be 1.25 wt% for bamboo. The observed discrepancies in ash content can be attributed to geographical origin, different species of bamboo, and harvest season. High mineral absorption for bamboo contributes to high levels of nitrogen and sulphur contents [41,42,43]. Hence, pine biomass is anticipated to emit minimal sulphur and nitrogen oxides during bio-oil synthesis, reinforcing its environmental compatibility over bamboo biomass. In the ultimate analysis, bamboo displays slightly lower carbon (48.97 wt%) and hydrogen (6.280 wt%) contents compared to pine (carbon: 51.16 wt%; hydrogen: 7.052 wt%). However, bamboo demonstrates a higher nitrogen content (0.280 wt%) compared to pine (0.043 wt%). Both bamboo and pine show minimal sulphur content, with bamboo slightly higher at 0.030 wt% compared to pine at 0.020 wt%. Bamboo appears to have a higher oxygen content (44.49 wt%) compared to pine (41.73 wt%). The high oxygen content of bamboo biomass correlated with its low heating value. These results indicated that bamboo contains more oxygenated compounds than pine biomass with a lower oxygen content. Considering calorific values, pine exhibits a higher (8% higher) calorific value (20.38 MJ/kg) compared to bamboo (18.70 MJ/kg), indicating differences in energy content per unit mass. Other biomass sources, such as coconut and cassava rhizomes, have HHVs of 17.77 and 23.67 MJ/kg, respectively [44]. Despite bamboo’s lower calorific value, its lower moisture content and higher fixed carbon content suggest it may still offer favorable characteristics during pyrolysis, particularly in terms of process efficiency and biochar yield. However, the higher volatile matter content in pine indicates a potentially higher bio-oil yield, making it a superior source for bio-oil production over bamboo.

### 3.2. Biomass Structural Composition

The biomass structural composition significantly influences the product distribution and quality of bio-oil during pyrolysis. The biomass structural composition data are tabulated in Table 2. Pine biomass exhibits lower water and ethanol extractives at 1.52 and 1.72 wt%, respectively, in contrast to bamboo with higher values of 6.15 and 1.88 wt%, respectively. These extractive results signify that bamboo biomass has the potential to yield more bio-oil aqueous phase than pine biomass. Despite the higher water extractives content in bamboo, which suggests higher bio-oil aqueous phase yields, the actual yields are influenced by various factors beyond extractives content. As previously stated, volatile content has a significant influence on bio-oil organic phase yield. Asadullah, Rahman [45] stated that volatile matter is converted to bio-oil upon condensation; hence, low volatiles for bamboo biomass support the prospect of low bio-oil yield. Other factors, such as the contents of lignin and carbohydrates (holocellulose), which affect reaction pathways during pyrolysis, play a significant role.

**Figure 2:**
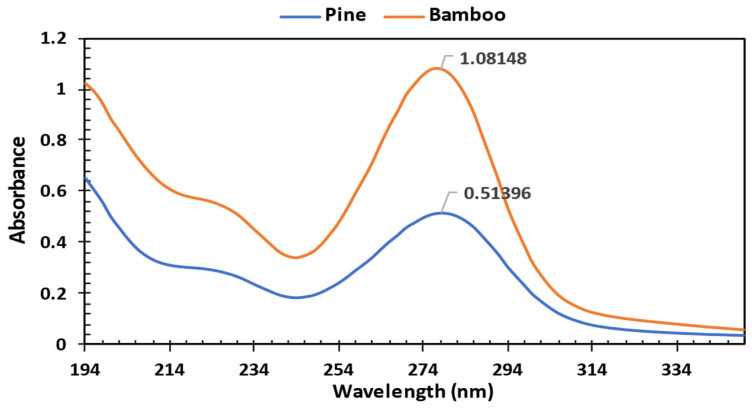
Absorbance peaks of pine and bamboo biomass after acid hydrolysis.

The UV/vis spectrophotometric analysis revealed distinct absorbance peaks for lignin in pine and bamboo biomass samples, with values of 0.51396 and 1.08148, respectively, at 280 nm (Figure 2). This wavelength corresponds to the typical absorbance range for lignin, attributed to its aromatic rings, which exhibit strong absorbance around 280 nm [46,47,48,49,50]. Although carbohydrates generally absorb in the 190–210 nm range [51,52,53], the current study specifically targeted lignin detection; hence, an absorbance range of 194–354 nm was selected. The significantly higher absorbance in bamboo suggests a relatively higher lignin content compared to pine, potentially impacting its degradation characteristics and suitability in biofuel applications. 

The Klason lignin content in bamboo (16.63 wt%) is associated with increased phenolic compound production in the bio-oil, making bamboo biomass favorable for bio-oil with phenolic compounds. Conversely, lower lignin content for pine biomass (9.37 wt%) may result in lower phenolic compound yields. The acid-soluble lignin (ASL) exhibits consistent values between pine and bamboo biomass with an absolute error of 1%. The carbohydrate profile for pine biomass includes glucose (43.50 wt%), xylose (35.47 wt%), galactose (16.04 wt%), arabinose (2.045 wt%), and mannose (2.063 wt%). Meanwhile, bamboo biomass exhibits glucose (43.07 wt%), xylose (32.86 wt%), galactose (15.51 wt%), arabinose (1.673 wt%), and mannose (1.690 wt%). The distribution of these components influences the composition of the resulting bio-oil. Different carbohydrates undergo varied thermal degradation pathways during pyrolysis, affecting bio-oil quality [54,55]. The glucose content was comparable among the two considered biomasses with an absolute error of 0.95% relative to pine. The mannose and galactose contents were marginally distinct, with an absolute error of 18% relative to pine. Xylose deviated slightly with an absolute error of 2.8% relative to pine, which signifies that xylose content in pine is 2.8% higher than that in bamboo. The overall estimates of lignin and holocellulose were 21.75 and 78.97 wt% for pine and 29.14 and 75.93 wt% for bamboo, respectively. Guiotoku, Pangrácio [43] carried out bamboo characterization, and the lignin and holocellulose values were closely correlated with the results of this study. Conversely, Kato, Kohnosu [40] reported Klason lignin of 24.37 wt% and holocellulose of 65.66 wt% for bamboo. This was attributed to harvest season, bamboo species, and geographical location. Ferreira-Santos, Genisheva [56] reported 29.7 wt% holocellulose and 41.05 wt% Klason lignin for pine. Correspondingly, Santos, Pereira [57] obtained 39.3 wt% holocellulose and Klason lignin of 46.2 wt% for pine. Hence, it is evident that the investigated pine species was rich in lignin. The “others” content, which includes proteins and lipids, was higher for bamboo biomass (5.2 wt%) in contrast to pine biomass (0.9 wt%). These results indicate that pine biomass can produce furans due to its high xylose content, while both biomasses may produce levoglucosan due to high glucose content.

### 3.3. Biomass FTIR

**Figure 3:**
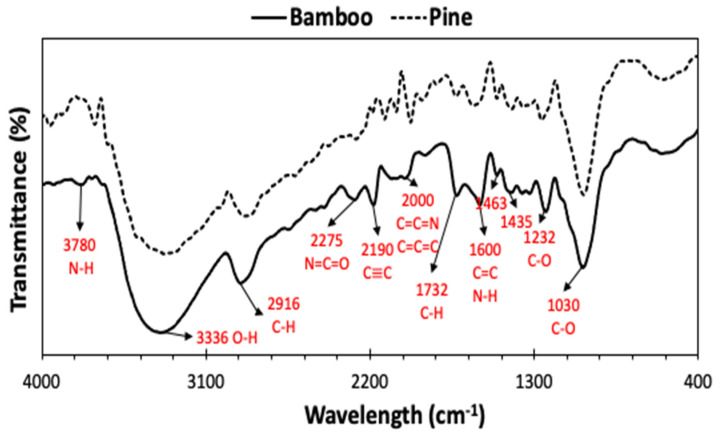
FTIR profiles of pine and bamboo biomass.

The categorization of biomass functional groups assists in understanding the chemical composition of the biomass sample, offering crucial insights for its analysis and utilization in thermochemical processes. Figure 3 illustrates the FTIR spectrum of biomass samples (bamboo and pine), revealing various functional groups through distinctive peaks. The broad peak observed at 3336 cm^−1^ denotes the O-H stretching vibrations of lignin and cellulose in the biomass. Similarly, the peaks at 2916 cm^−1^ signify the C-H stretching vibrations, indicating the presence of aliphatic compounds in the hemicellulose [58,59]. The peak at 1732 cm^−1^ originates from the C-H and C=O stretching vibration of oxygen double bonds in hemicellulose, which is also contributed to by lignin [60]. Aldehyde functionalities, mainly derived from xylose, are indicated by 1435 and 1463 cm^−1^ peaks, which may also relate to N-H bending, C=C stretching, and C-H bending vibrations. Bamboo biomass exhibits a significant presence of nitrogenous compounds compared to pine biomass, as seen in Figure 3. The peaks for these compounds were prominent in the bamboo biomass. Peaks observed between 1200 and 1600 cm^−1^ suggest O-H bending and S=O stretching resonances, hinting at carboxylic acid and sulfonate/phenol functionalities in the biomass. The peak at 1232 cm^−1^ signifies C-O stretching vibrations of lignin and xylan, along with syringyl ring breathing. The peak at 1030 cm^−1^ is associated with stretching vibrations of S=O/C-N functionalities (inorganics in biomass). Additionally, peak 1030 cm^−1^ was also attributed to the presence of cellulose I crystalline and C-O bonding [61,62].

### 3.4. Fast Pyrolysis Product Distribution

**Figure 4:**
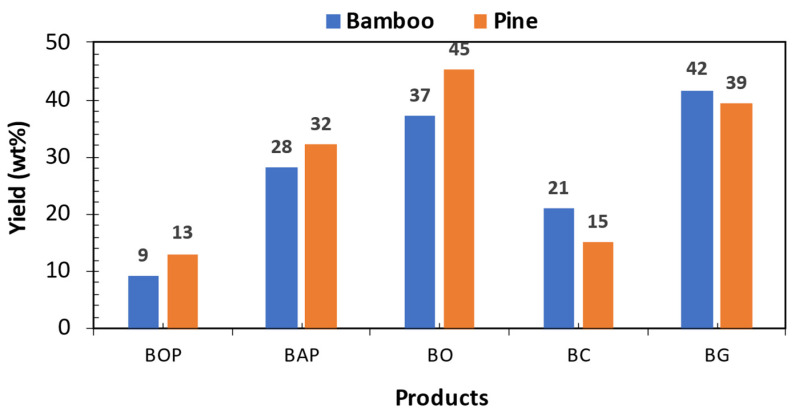
Fast pyrolysis product yields [BOP: bio-oil organic phase, BAP: bio-oil aqueous phase, BO: bio-oil, BC: biochar, and BG: pyrolysis gas].

The influence of different biomass (pine and bamboo) on the pyrolysis product distribution (biochar, bio-oil, and gas) was examined and plotted in Figure 4. Pine and bamboo biomass were subjected to pyrolysis at 550 °C to examine the effect of woody and cane-based biomass on product distribution. Moreover, bio-oil yield can only be accurate if the two phases are quantified separately. In the present study, bio-oil phases were quantified separately. It is observed that the bio-oil organic phase (BOP) yield was higher for pine biomass at 13 wt% in contrast to bamboo biomass (9 wt%). The bio-oil yields for pine and bamboo were 45% and 37% by weight, respectively. In a study by Varma and Mondal [14], a bio-oil yield of 43.76% was achieved at 550 °C from pinewood biomass. Correspondingly, Chen, Liu [63] reported a bio-oil yield of 36.6% by weight at 500 °C from bamboo biomass. These findings align closely with the trends observed in the current study.

**Figure 5:**
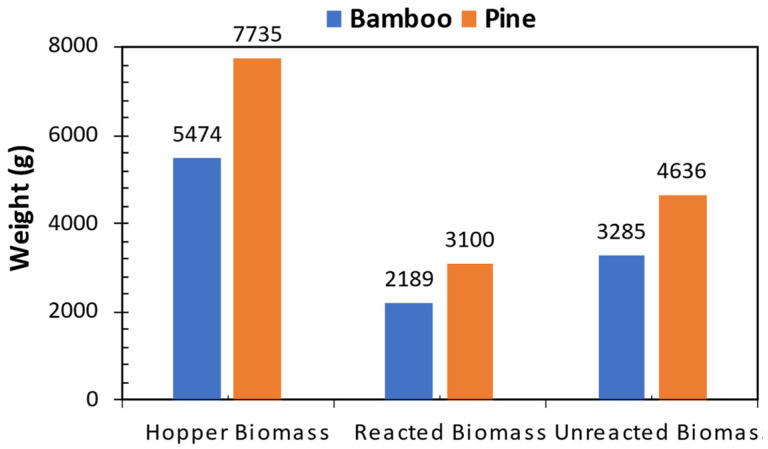
Biomass balance for fast pyrolysis in a fluidized bed reactor.

The primary decomposition reactions of the pine and bamboo biomasses were somehow comparable, with the only distinction arising from the reacted biomass. The mass difference between the initial (hopper biomass) and the final (unreacted biomass) was classified as reacted biomass. The fibrous nature of bamboo biomass resulted in less-reacted biomass at constant reaction time due to flow resistance during pyrolysis (Figure 5). Bamboo pyrolysis is subjected to high operating costs due to prolonged reaction times, leading to higher energy utilization. Bamboo biomass resulted in biochar and pyrolysis gas yields of 21 and 42 wt%, respectively, at low reacted biomass, which correlated to high fixed carbon content and extractives. These outcomes suggest that bamboo pyrolysis results in continued cracking of the products at a temperature of 550 °C, as indicated by higher non-condensable gas yields of CO_2_, CO, and CH_4_ [64] (Figure 4). A similar trend was noted by Fahmi, Bridgwater [65], who attributed it to the presence of inorganic compounds (ash) in the biomass. The authors found that higher inorganic content led to a reduction in bio-oil yield, accompanied by increased yields of biochar and pyrolysis gases. In this study, as shown in Table 1, the ash content of pine biomass is approximately 83% lower than that of bamboo, which accounts for the observed lower biochar and pyrolysis gas yields for bamboo.

### 3.5. BOP Characterization: Ultimate, and Properties

The analyses of bio-oil samples collected during the thermal degradation of bamboo and pine biomass are tabulated in Table 3. These analyses exhibited discrepancies in both ultimate analysis and properties of BOP. Moisture content showed a slight variation, with bamboo displaying the higher value at 9.82 ± 0.135 wt% with an absolute error of 0.3% relative to pine moisture content. The observed high moisture content for bamboo bio-oil coincides with the high yield of the aqueous phase (Figure 4). Ash content refers to the inorganic material left behind after the combustion of a fuel. In this study, the ash content in BOPs ranged from 0.029 wt% for pine to 0.21 wt% for bamboo. These results suggest that bamboo BOPs contain a higher level of inorganic material compared to pine BOP. The difference in ash content is attributed to bamboo’s greater ability to absorb minerals, resulting in higher inorganic concentrations. Furthermore, the data show that higher ash content in biomass leads to an increase in ash content in the bio-oil organic phase. Elevated ash levels in liquid fuels like bio-oil can cause several operational issues, including increased wear on pumps and injectors, as well as the formation of deposits and corrosion in combustion systems. These problems are mainly due to the presence of alkali metals in the ash.

The ultimate analysis exhibits significant differences in the carbon, nitrogen, sulphur, and oxygen contents for pine and bamboo organic phase bio-oils. The hydrogen content, on the other hand, remained relatively constant with an absolute error of 1.1%. Bamboo bio-oil exhibited the highest carbon content at 70.42 ± 0.75 wt%, while pine bio-oil showed the highest oxygen content at 27.42 ± 0.55 wt%. The carbon content of pine bio-oil is 8.3% lower than that of bamboo. Meanwhile, the oxygen content of bamboo bio-oil is 23% lower than that of pine bio-oil. Considering the 0.3% absolute error in the moisture content, it is evident that pine bio-oil contained more oxygenated compounds than bamboo bio-oil. Pine bio-oil exhibits lower nitrogen content (0.18 ± 0.07 wt%) than bamboo bio-oil (0.54 ± 0.02 wt%). These results suggest that pine bio-oil has a lower tendency to produce air-polluting impurities, such as nitrogen oxides. The sulphur content is lower for bamboo bio-oil at 0.02 ± 0.02 wt% and 50% higher for pine bio-oil. The high nitrogen content for bamboo bio-oil was attributed to nutrient absorption by bamboo plants [66,67], considering the significance of nitrogen for plant growth, especially for cane-based biomass (energy crops). The properties of the bio-oil samples, i.e., kinematic viscosity, density, total acid number (TAN), dynamic viscosity, and higher heating value (HHV), also varied for pine and bamboo bio-oils. Pine bio-oil demonstrated higher TAN and viscosity values at 63.85 ± 2.45 mgKOH/g and 67.85 ± 1.25 mm^2^/s, respectively, compared to bamboo bio-oil at 54.13 ± 1.24 mgKOH/g and 53.25 ± 1.15 mm^2^/s, indicating differences in acidity levels as well as flow characteristics. The cane-based bio-oil exhibits lower acidity when compared to wood-based bio-oil. This was attributed to different structural compositions in biomass, which influences the bio-oil quality [54]. Meanwhile, the density of pine bio-oil is 1.18 g/cm^3^, whereas the density of bamboo bio-oil showed a 3.4% deviation from the pine bio-oil. The density values were somewhat close to the ones reported in the literature for grape bagasse [68], coconut shell [69], cassava rhizome [70], rice husk [71], and palm tree [72]. The HHV values exhibited a higher value for pine bio-oil (23.90 ± 0.06 MJ/kg) when compared to bamboo bio-oil (22.89 ± 0.04 MJ/kg), suggesting variations in the energy content of the bio-oil samples. The observed disparity between the heating values was insignificant, as indicated by the absolute error of 4.2% relative to pine bio-oil. In a study conducted by Hassan, Steele [73], the authors reported a density of 1.18 g/cm^3^ and an HHV of 22.49 MJ/kg for bio-oil derived from pine biomass. These values are consistent with the findings obtained in the present study. Khuenkaeo and Tippayawong [74] reported an HHV of 20.24 MJ/kg for bio-oil from bamboo residues, which contrasts with the value obtained in this study. This discrepancy is likely due to differences in biomass characteristics, operating conditions, and the type of pyrolysis reactor used. Cane-based bio-oil showed improved fuel properties when compared to wood-based bio-oil. These improvements, however, come with trade-offs, such as high inorganics in the cane-based biomass, which will result in higher ash content in the bio-oil. Moreover, the presence of impurities such as sulphur in the bio-oil is advantageous during hydrotreating when using sulphided catalysts [75,76]. This will negate the need for an external sulphur source, such as hydrogen sulphide, in order to keep the catalyst active. Hence, the choice of bio-oil source is somehow dependent on the chosen method of bio-oil stabilizing and upgrading techniques.

### 3.6. Bio-Oil Organic Phase GCMS

**Figure 6:**
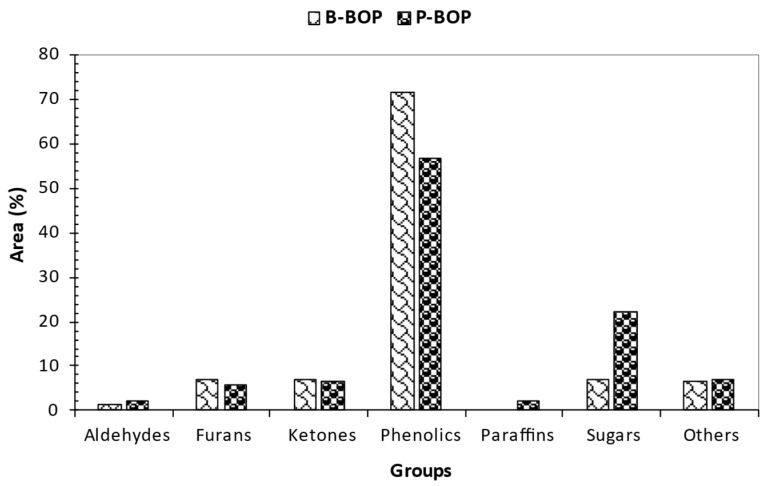
GCMS analysis of bio-oil organic phase for pine (P-BOP) and bamboo (B-BOP).

To assess the distribution of products across different samples, the compounds identified via GCMS were categorized into six groups based on their functional groups: phenols, esters, ketones, aldehydes, sugar derivatives, and furans. Figure 6 summarizes the proportional amounts of compound classes in the bio-oil organic phase (BOP). Each group’s total proportional area (%) was determined by summing each compound’s proportional area (%) within that category.

Pyrolysis incorporates complex reactions owing to the degradation of hemicellulose, cellulose, and lignin. Decomposition of cellulose and hemicellulose forms 1-hydroxy-2-propanone, acetic acid, anhydro sugars, furans, and levoglucosan, among other compounds. Meanwhile, phenolics, aldehydes, ketones, guaiacols, alcohols, and carboxylic acids come from lignin decomposition [77]. Phenolic compounds were prominent groups in the bio-oil for both pine and bamboo biomass. However, bamboo exhibited higher phenolics with an absolute error of 21% relative to pine phenolics. These results were attributed to higher lignin content for bamboo than pine biomass (Table 2). Bio-based phenols could be used as renewable resins when extracted and potentially replace conventional petroleum-based phenols. Ketones are present in both biomass at consistent proportions. These compounds tend to form esters when oxidized. Phenolics and ketones are oxygenated compounds that contribute significantly to lowering the heating value of bio-oil [78]. Moreover, phenolics and furans are typical products of cellulose and lignin decomposition [79]. Acid compounds (classified under “others” in Figure 6) and their derivatives are formed due to the decomposition of hemicellulose. The nitrogenous compounds were attributed to the heterocyclic ring-containing compounds in the biomass [80]. Bamboo biomass showed the presence of amines, which was attributed to strong nutrient absorption during plant growth. Amines are not desirable as fuel; however, amine solvents are essential in absorbing carbon dioxide from flue gas via amine-based post-combustion capture (PCC), thus preventing air pollution. Pine exhibited high sugar content when compared to bamboo. The main sugar compound that was obtained in bio-oils was levoglucosan from the decomposition of cellulose. It is formed during a depolymerization reaction due to transglycosylation at a temperature of 300 °C [16]. The observed decrease in sugars for bamboo was attributed to fission and disproportionation, which leads to the formation of furans and acids. Additionally, the high content of inorganics in bamboo, which promotes glucose fragmentation rather than polymerization, also contributes to sugar compound reduction. 1,4:3,6-Dianhydro-α-d-glucopyranose, the dehydrated form of levoglucosan, and D-Allose were also obtained in bio-oils. This signified that the constituent structure of biomass highly influences the distribution of pyrolysis products [16].

### 3.7. Bio-Oil Organic Phase FTIR

**Figure 7:**
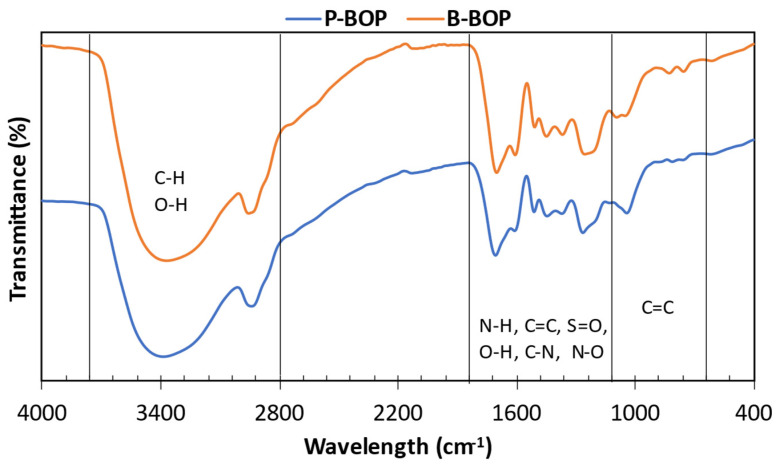
FTIR spectra of bio-oil organic phase for pine (P-BOP) and bamboo (B-BOP).

FTIR spectroscopy, as outlined by Lievens, Ci [74], serves as a potent analytical tool for elucidating the functional groups present in pyrolysis bio-oils. The infrared spectra of bio-oil samples from pine and bamboo biomass after thermal degradation are shown in Figure 7. Analysis of the infrared spectra of bio-oil samples derived from pine and bamboo biomass reveals that both types of biomass yield bio-oils containing similar functional groups. However, it is noteworthy that bamboo biomass may contain more inorganic compounds due to its higher nutrient absorption during growth. Spectral analysis indicates the presence of key functional groups, including C=O, C=C, C–O, C–H, and O–H bonds, within the spectral ranges of 3000–3500 cm^−1^ and 800–1750 cm^−1^. These functional groups suggest the presence of alcohols, phenols, aromatics, and acids in the bio-oil, which aligns with findings from GC–MS analysis. Furthermore, the FTIR spectra in the range of 1490–1850 cm^−1^ offer detailed insights into various carbonyl groups present in the bio-oil, as reported by Lievens, Ci [81]. Considering that biomass mainly comprises CHO chemical compounds derived from cellulose, hemicellulose, and lignin [82], these results underscore the potential for producing value-added products through the pyrolysis of pine and bamboo biomass. Moreover, the organic phase of the bio-oil presents promising opportunities for enhancement through hydrotreatment.

### 3.8. Biochar Characterization

Table 4 indicates the proximate and ultimate analysis of the biochar for bamboo and pine after the thermal degradation process in the fluidized bed reactor. The proximate and ultimate analyses of bamboo and pine biomass subjected to pyrolysis in a fluidized bed reactor yield valuable insights into their respective biochar characteristics. Bamboo exhibits a lower moisture content at 0.174 wt% than pine (0.014 wt%), indicating a potentially higher efficiency due to reduced energy requirements for moisture removal. However, such a low moisture content could potentially eliminate the requirement for supplementary drying, contingent upon compliance with specified biochar quality standards. Pine demonstrates a higher volatile content at 17.27 wt% compared to bamboo (13.52 wt%), suggesting greater combustible matter in pine biochar. Additionally, bamboo biochar contains significantly higher ash content (74.07 wt%) compared to pine (55.56 wt%), owing to variations in mineral composition. Pine biochar exhibits superior fixed carbon content (27.17 wt%) compared to bamboo at 12.41 wt%, indicating a higher potential for stable carbon sequestration. Although both biochars exhibit almost comparable carbon content, pine biochar demonstrates marginally higher hydrogen (2.658 wt%) and lower oxygen (29.47 wt%) content, contributing to its slightly elevated calorific value (24.24 MJ/kg) compared to bamboo biochar (23.03 MJ/kg). Therefore, considering its lower moisture content, higher volatile matter, and favorable fixed carbon content, pine biomass emerges as the preferred choice for biochar production in fluidized bed reactor pyrolysis processes. Furthermore, the biochar produced has substantial potential for generating heat and power through boilers, gasifiers, and furnaces. Additionally, it offers diverse applications, including its use as fertilizer, activated carbon, and for enhancing catalytic processes, as cited in references [83,84,85,86,87,88,89,90].

### 3.9. Van Krevelen Plot

**Figure 8:**
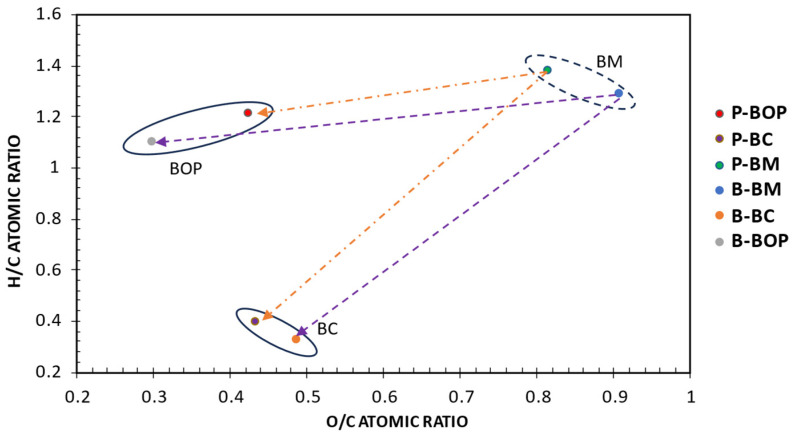
Van Krevelen plot of pine (P) and bamboo (B) for biomass (BM), biochar (BC), and bio-oil organic phase (BOP).

The atomic ratios of hydrogen/carbon (H/C) and oxygen/carbon (O/C) of pine (P) and bamboo (B) biomass (BM), biochar (BC), and bio-oil organic phase (BOP) were evaluated and compared as shown in the Van Krevelen diagram in Figure 8. This plot tracks changes in the molecular composition of feedstocks during pyrolysis, offering insight into the evolution of product distribution and the degree of aromaticity in the material [91]. In pyrolysis, the van Krevelen plot is often used to understand the progression from biomass or other organic materials to pyrolytic products, such as bio-oils and pyrolysis gases. It shows how the H/C and O/C ratios change as a function of temperature and reaction conditions. Van Krevelen [91] first discussed the concept in relation to the combustion and pyrolysis of organic materials, particularly coals. The plot in Figure 8 highlights three distinct regions for biomass, biochar, and bio-oil organic phase, with the biomass at the top right corner and the bio-oil organic phase at the lower left corner. Higher H/C ratios are desired for the fuel to be more efficient through the reduction in CO emissions. Pine bio-oil resulted in higher H/C and O/C ratios, while bamboo bio-oil exhibited lower H/C and O/C ratios. These results imply that pine bio-oil is more efficient in reducing CO emissions during bio-oil stabilization and hydrotreatment. Pine biochar exhibited higher H/C and lower O/C ratios. Meanwhile, bamboo bio-oil exhibited higher O/C and lower H/C ratios. The decreased H/C ratios observed in the pine and bamboo biochar samples suggest that higher levels of aromatic compounds were formed in the biochar structures due to hydration, decarboxylation, and decarbonylation during processing at the operating temperature. Consequently, this process enriched the biochar with carbon, resulting in a surface that is highly resistant to water [90,92,93,94].

### 3.10. SEM/EDX of Biochar

**Figure 9:**
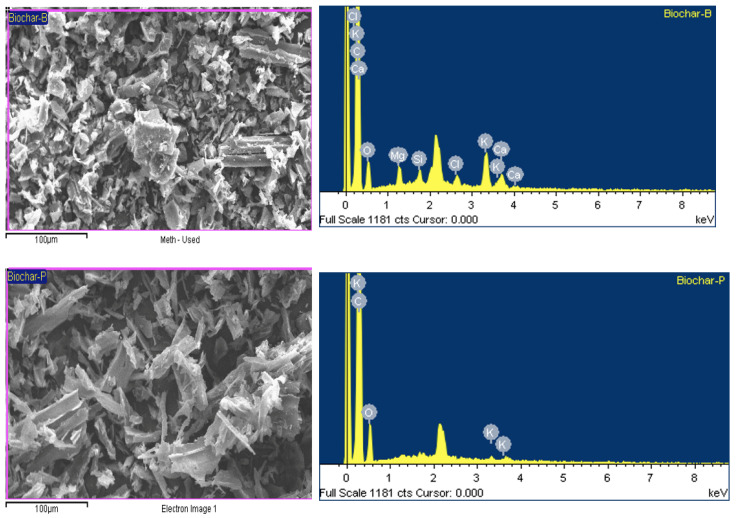
SEM/EDX of bamboo biochar (biochar-B) and pine biochar (Biochar-P).

The SEM/EDX analysis (Figure 9) of biochar derived from bamboo and pine biomass demonstrates distinct differences in their elemental compositions, which have significant implications for their respective applications. Bamboo biochar exhibits a diverse range of elements with the presence of carbon (C), oxygen (O), potassium (K), calcium (Ca), magnesium (Mg), silicates (Si), and traces of chlorine (Cl). The substantial carbon content confirms effective carbonization during pyrolysis, while the diverse mineral content, including high potassium, calcium, and magnesium levels, indicates a significant ash component [95]. This composition suggests that bamboo biochar has enhanced capabilities for soil amendment, including improved pH buffering, nutrient supply, and potential soil fertility benefits due to calcium’s liming effects and potassium and magnesium’s fertilizing properties [14,96]. Applying biochar to soil resulted in mineral phases adhering to its surface, which made the elemental composition more complex. This effect is likely due to the biochar’s carboxylic and phenolic functional groups, which can bond with multi-valent cations such as Si^2^⁺, Al^3^⁺, and Fe^3^⁺, creating organo–mineral complexes. These organo–minerals typically boost soil nutrient transformation and adsorption. Biochar with finer particles and high internal porosity is expected to improve soil quality significantly [97]. The presence of silicates may further enhance the biochar’s mechanical strength and stability. In contrast, pine biochar contains primarily carbon, oxygen, potassium, and hydrogen, with fewer mineral elements. The carbon content indicates successful pyrolysis, but the reduced presence of calcium, magnesium, and silicate limits its potential as a multifunctional soil conditioner. SEM/EDX analysis revealed that the surface of biochar is rich in various nutrients. It can serve as an adsorbent in water purification processes and enhance soil fertility. Additionally, because of its high pH, biochar is effective for soil amendment, helping to neutralize soil acidity and raise the soil pH. Pine biochar’s more straightforward mineral composition may be preferable in applications requiring minimal alteration of soil mineral content, while bamboo biochar’s broader mineral profile positions it as a more versatile option for comprehensive soil and environmental management strategies.

### 3.11. Energy Yield and Gas Analysis

**Figure 10:**
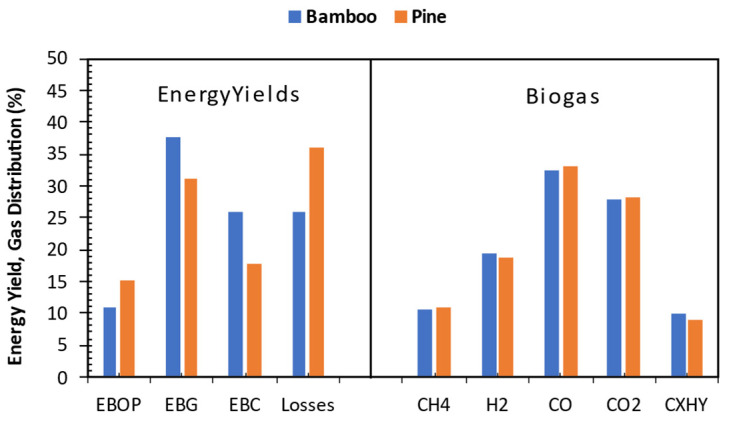
Biogas analysis and energy yields for bio-oil organic phase (EBOP), biogas (EBG), biochar (EBC), and losses.

The gas compositions, including hydrogen (H_2_), methane (CH_4_), carbon monoxide (CO), carbon dioxide (CO_2_), and others (C_x_H_y_), along with the energy yields resulting from thermal degradation experiments on pine and bamboo biomass, are depicted in Figure 10. Methane production is attributed to lignin deformation occurring at the operating pyrolysis temperature. The methane yield is 10.5% for bamboo and 11% for pine. Moreover, cellulose composition features a significant presence of the –HCOH segment, the decomposition of which could yield -H and -CO or -CH and -OH [98]. Combining -CH with numerous -H atoms produces CH_4_, while CO_2_ forms by combining -CO with -OH or an oxygen radical. Studies indicate that lignin deformation and cracking release more hydrogen and methane compared to cellulose and hemicellulose [99,100,101,102,103,104]. Furthermore, the water gas shift reaction is also an attribute to the formation of hydrogen and carbon dioxide. This is because cellulose contains more OH and C–O compounds, hemicellulose comprises higher levels of C=O organic compounds, whereas lignin is rich in aromatic rings and O–CH_3_ functional groups. Gas analysis reveals that carbon monoxide is the primary compound in bamboo and pine pyrolysis gases at 550 °C (32.5% and 33.1%, respectively). The reverse water gas reaction (H_2_ + CO_2_ = CO + H_2_O) may occur, resulting in a more significant generation of CO while reducing the quantities of H_2_ and CO_2_ [105]. Carbon dioxide, regarded as an inert gas (excluded in the biogas energy content), is the second highest component at 27.8% for bamboo and 28.3% for pine, likely due to decarbonylation and decarboxylation reactions increasing CO levels. Cracking the bond between the C1 carbon atom and two oxygen atoms in the gluconic unit of cellulose may produce CO_2_ [106]. The substantial presence of hydrogen (19.3% for bamboo and 18.75% for pine) in the biogas indicates its potential as a hydrogen source for downstream bio-oil hydrotreating to meet fuel standards. The dehydrogenation reactions result in the formation of H_2_. Furthermore, the breakdown of lignin releases hydrogen gas as some of its structures decompose [107].

It is observed that the pine energy yield for the bio-oil organic phase (EBOP) is higher than that of bamboo, at 15% and 11%, respectively. This can be explained by the better energy value and higher oil production for pine biomass (Figure 10). Biogas had the highest energy fraction among the product energy yields, with bamboo and pine biogas at 38% and 31%, respectively. The second highest energy yield was observed for biochar, with bamboo at 26% and pine at 18%. The yields of the pyrolysis products correlated with the observed energy yields despite biogas having the lowest heating values and higher yields. The determined heating values for biogas were 16.76 and 16.35 MJ/kg for bamboo and pine, respectively. It is worth noting that bio-oil organic phase energy yield did not incorporate the aqueous phase, which is usually quantified as bio-oil yield. During the continuous pyrolysis process, some organic bio-oil ends up in the aqueous phase. For this work, only the pure organic phase, i.e., ESP oil, was considered. The trade-off to the aforementioned procedure is the addition of losses. The observed energy losses were from 26% to 36% for bamboo and pine, respectively. Bamboo most efficiently converted 74% of the feedstock energy into pyrolysis products. The observed losses were primarily due to the consideration of organic bio-oil only. Additionally, some organic bio-oil couldn’t be retrieved from the condensing unit due to the complexities involved in collecting all the resulting liquid yield. Consequently, a portion of bio-oil energy was lost, impacting the process’s efficiency in terms of energy yields.

## 4. Conclusions

A comparative study employing isothermal fast pyrolysis in a sand-fluidized bed reactor investigated bamboo and pine biomass for the production and characterization of bio-oil and associated by-products. Pine bio-oil exhibited superior energy density compared to bamboo bio-oil, indicating variations in their energy content. Consequently, pine bio-oil emerges as more favorable for commercialization, considering bamboo’s higher flow resistance during fluidized bed reactor pyrolysis and the higher heating value (HHV) of pine bio-oil. Bio-oils are viable for fuel applications and chemical production following refining and enhancement processes. Bamboo bio-oil displayed lower acidity, attributed to differing biomass structural compositions. Pine bio-oil demonstrated higher H/C and O/C ratios, whereas bamboo bio-oil exhibited lower ratios, suggesting pine bio-oil’s potential efficacy in reducing CO emissions during stabilization and hydrotreatment. Spectroscopic and chromatographic analyses revealed numerous oxygenated compounds in the bio-oils. With its elevated carbon content and HHV of 24.24 MJ/kg, pine biochar holds promise for energy applications and as a precursor in activated carbon production. Gaseous products included carbon monoxide and hydrogen, which are suitable for fuel applications or conversion into liquid fuels, with hydrogen particularly valuable for bio-oil upgrading. Pine biomass yielded a higher energy yield for the bio-oil organic phase (EBOP) than bamboo, at 15% and 11%, respectively, attributable to superior energy content and enhanced oil yield from pine biomass. The study concludes that pine and bamboo biomass are viable renewable energy sources, contingent upon efficiently utilizing all pyrolysis products.

## Figures and Tables

**Table 1:** Proximate and ultimate analysis of lignocellulosic biomass.

Parameter	Bamboo	Pine
Proximate Analysis (wt%) ^db^		
Moisture content ^a^	4.010 ± 0.13	6.760 ± 0.28
Volatile content	85.90 ± 0.70	92.50 ± 0.60
Ash content	1.970 ± 0.02	0.330 ± 0.07
Fixed carbon ^b^	12.13	7.17
Ultimate Analysis (wt%) ^db^		
Carbon	48.97 ± 0.170	51.16 ± 0.130
Hydrogen	6.280 ± 0.180	7.052 ± 0.160
Nitrogen	0.280 ± 0.047	0.043 ± 0.011
Sulphur	0.030 ± 0.009	0.020 ± 0.010
Oxygen	44.49 ± 0.160	41.73 ± 0.010
HHV (MJ/kg)	18.70 ± 0.045	20.38 ± 0.054

^db^ dry basis, ^b^ calculated by difference, ^a^ wet basis.

**Table 2:** Structural composition of pine and bamboo biomass.

Components	Pine	Bamboo
Extractives (wt%) ^db^		
Water	1.52 ± 0.05	6.15 ± 0.03
Ethanol	1.72 ± 0.02	1.88 ± 0.04
Lignin (wt%) ^db,e^		
Klason (AIL)	9.367 ± 0.04	16.63 ± 0.05
Acid soluble lignin (ASL)	12.38 ± 0.07	12.51 ± 0.03
Carbohydrates (wt%) ^db,e^		
Glucose	43.50 ± 0.03	43.07 ± 0.09
Xylose	35.47 ± 0.08	32.86 ± 0.05
Galactose	16.04 ± 0.02	15.51 ± 0.01
Arabinose	2.045 ± 0.17	1.673 ± 0.23
Mannose	2.063 ± 0.13	1.690 ± 0.11

^db^ dry basis, ^e^ extractive free.

**Table 3:** Ultimate, and properties of bio-oil organic phase.

Parameter	Bamboo	Pine
Ultimate Analysis (wt%) ^db^		
Carbon	70.82 ± 0.75	64.54 ± 0.45
Hydrogen	7.910 ± 0.08	7.820 ± 0.02
Nitrogen	0.540 ± 0.02	0.180 ± 0.07
Sulphur	0.020 ± 0.02	0.040 ± 0.02
Oxygen	21.11 ± 0.67	27.42 ± 0.55
C/H	9.00 ± 0.05	8.252 ± 0.82
H/C	0.11 ± 0.00	0.121 ± 0.01
O/C	0.30 ± 0.19	0.425 ± 0.22
Properties		
Moisture content (wt%) ^a^	9.82 ± 0.135	9.79 ± 0.180
Ash content (wt%) ^db^	0.210 ± 0.01	0.029 ± 0.00
K. viscosity (mm^2^/s) ^ar^ 40 °C	53.25 ± 1.15	67.85 ± 1.25
Density (g/cm^3^) ^ar^ 40 °C	1.14 ± 0.00	1.18 ± 0.00
Dynamic viscosity (mPa⋅s) ^ar^	60.71 ± 1.31	80.06 ± 1.47
TAN (mgKOH/g) ^ar^	54.13 ± 1.24	63.85 ± 2.25
HHV (MJ/kg) ^db^	22.89 ± 0.04	23.90 ± 0.06

^a^ wet basis, ^db^ dry basis, ^ar^ as received.

**Table 4:** Proximate, ultimate, and calorific values of biochar.

Parameter	Bamboo	Pine
Proximate Analysis (wt%) ^db^		
Moisture content ^a^	0.174 ± 0.160	0.014 ± 0.002
Volatile content	13.52 ± 0.233	17.27 ± 0.243
Ash content	74.07 ± 1.597	55.56 ± 1.821
Fixed carbon ^b^	12.41	27.17
Ultimate Analysis (wt%) ^db^		
Carbon	65.55 ± 3.12	67.76 ± 6.10
Hydrogen	2.134 ± 0.13	2.658 ± 0.18
Nitrogen	0.401 ± 0.04	0.127 ± 0.01
Sulphur	0.036 ± 0.04	0.007 ± 0.01
Oxygen	32.06 ± 2.85	29.47 ± 6.70
O/C	0.488 ± 0.02	0.430 ± 0.06
H/C	0.033 ± 0.00	0.039 ± 0.00
Calorific value (MJ/kg) ^db^	23.03 ± 0.01	24.24 ± 0.02

^db^ dry basis, ^a^ wet basis, ^b^ determined by difference.

## Data Availability

The data presented in this study are available on request from the corresponding author.
